# Dietary Therapy to Improve Nutrition and Gut Health in Paediatric Crohn’s Disease; A Feasibility Study

**DOI:** 10.3390/nu14214598

**Published:** 2022-11-01

**Authors:** Stephen J. Allen, Salma Belnour, Elizabeth Renji, Bernie Carter, Lucy Bray, Angela Allen, Emma Jones, Britta Urban, Sarah Moule, Duolao Wang, Raymond J. Playford

**Affiliations:** 1 Department of Clinical Sciences, Liverpool School of Tropical Medicine, Pembroke Place, Liverpool L3 5QA, UK; 2 Alder Hey Children’s NHS Foundation Trust, Eaton Road, Liverpool L12 2AP, UK; 3 Department of Health Sciences, University of Liverpool, Foundation Building, Brownlow Hill, Liverpool L69 7ZX, UK; 4 Faculty of Health, Social Care and Medicine, Edge Hill University, St Helens Road, Ormskirk L39 4QP, UK; 5 Independent Researcher, Hartlepool TS27 4RR, UK; 6 School of Biomedical Sciences, University of West London, St Mary’s Road, Ealing, London W5 5RF, UK; 7 School of Medicine, College of Medicine and Health, University College Cork, College Road, T12 K8AF Cork, Ireland

**Keywords:** children, young people, Crohn’s disease, randomised trial, bovine colostrum, acceptability, quality of life, biomarkers

## Abstract

Bovine colostrum (BC) has anti-inflammatory, anti-infective, growth and intestinal repair factors that may be beneficial in Crohn’s disease (CD). We assessed whether daily BC for up to 3 months was acceptable to children and young people (CYP) with CD in remission or of mild/moderate severity. CYP were randomised to receive either BC or matching placebo milk daily for 6 weeks (blinded phase); all received BC for the following 6 weeks (open phase). In 23 CYP, median (inter-quartile range) age was 15.2 (13.9–16.1) years and 9 (39.1%) were girls. A similar proportion of CYP in the BC and placebo arms completed the blinded phase (8/12, 75.0% and 9/11, 81.8% respectively). Twelve (70.6%) CYP completed the open phase with 7 (58.3%) tolerating BC for 3 months. Diaries in weeks 2, 6 and 12 revealed that most CYP took BC every day (5/7, 71.4%; 5/8, 62.5% and 6/11, 54.5% respectively). In interviews, opinions were divided as to preference of BC over the placebo milk and some preferred BC over other nutritional supplements. Symptoms, clinical and laboratory variables and quality of life were similar in the two arms. BC may be an acceptable nutritional supplement for daily, longer-term use in CYP with CD.

## 1. Introduction

Crohn’s disease (CD), an incurable, chronic inflammatory bowel disease, results from a complex interplay between host genetics, environmental factors and the intestinal microbiota with abnormalities in mucosal barrier function and innate and adaptive immune responses [1]. Up to 25% of new diagnoses are made in children and young people (CYP) [2]. Recommended first line treatment for CYP with low-risk luminal CD is with exclusive enteral nutrition (EEN) for 6–8 weeks using a Food for Special Medicinal Purpose (FSMP) [3] and this results in remission or improvement in up to 90% of CYP [4,5]. However, many have persisting intestinal inflammation despite clinical remission [6] and disease flares occur in the majority of children within 12 months of re-introduction of their usual diet [4,7]. 

Continuing a FSMP alongside a normal diet improves nutrition and reduces relapses in both adults and children [8,9]. However, acceptability of FSMP for long-term use is limited by their poor palatability, and reluctance to have a naso-gastric tube [10,11]. In some adult studies, the taste or smell of feeds was reported to cause intolerance [8].

Bovine colostrum (BC), a commercial side product of the dairy industry, is produced by cows for the first few days after parturition and contains antimicrobial, immunomodulatory, and growth-stimulating factors at much higher concentrations than mature milk [12]. Multiple in vitro and in vivo studies indicate that BC reduces intestinal inflammation and improves mucosal integrity for several gastrointestinal disorders [12,13]. There are, however, only limited data from clinical trials. We assessed the acceptability of BC in paediatric CD and generated pilot data on its effects on disease symptoms, biomarkers of intestinal integrity and inflammation and quality of life.

## 2. Materials and Methods

### 2.1. Public and Public Involvement

Prior to ethics approval, we engaged 10 CYP with CD under the clinical care of the Paediatric Gastroenterology Department at Alder Hey Children’s Hospital, Liverpool, UK and their parents/carers in an activities-based workshop that explored the key issues underpinning the research. This enabled us to refine our explanations of key study-related concepts (e.g., dietary supplements; nutraceuticals), examine CYPs’ interest/lack of interest in and their perceptions of dietary therapy, their likelihood of participating in a study and the associated procedures including sample collection. We also obtained feedback on the design and content of consent/assent forms, information sheets and the format of a daily adherence and symptom diary and identified potential CYP and/or parent-centred STOP and GO markers. We also liaised with the Alder Hey GenerationR Young Persons’ Advisory Group to identify a young person with CD to advise on specific issues raised during trial set-up and as the trial progressed.

### 2.2. Intervention Trial

We then proceeded to a prospective, randomized, controlled interventional and qualitative feasibility study which recruited CYP aged 8–17 years with CD attending Alder Hey. CD was of mild/moderate severity (weighted pCD Activity Index [wPCDAI] ≤ 57.5) [14] and stable (receiving no drug treatment or no change in treatment for the last 2 months and no intention to change treatment). CYP were excluded if they were unwilling to discontinue a FSMP, were intolerant of dairy products, had a significant gut disorder other than CD or had insufficient knowledge of English to complete symptom diaries.

We reviewed clinical records and CYP who were likely eligible were sent by post an age-appropriate Patient Information Sheet (8–10 years, 11–15 years, 16–17 years and parents/guardians). CYP could express an interest in participating during a follow-up call or during their next hospital attendance or by contacting the research nurse. Informed consent was secured from CYP aged 16–17 years or from the parents/guardians of younger children. 

In the initial blinded phase (1–6 weeks), CYP were allocated 1:1 to the study arms according to a computer-generated, random allocation sequence with blocks of varied size generated and held by the Tropical Clinical Trials Unit (TCTU) at LSTM and not available to any member of the research team until after the final database was locked. Research staff independent of the research team prepared bags containing either BC or placebo milk powder labelled with the unique study number according to the allocation sequence but otherwise of identical appearance. Research nurses allocated each CYP to the next number in the sequence and provided the corresponding labelled bag.

**Figure 1:**
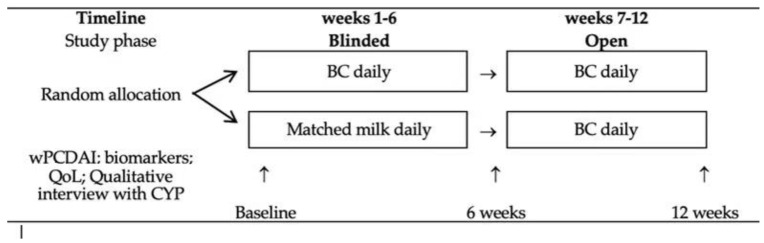
Trial design. BC = bovine colostrum; wPCDAI = weighted paediatric Crohn’s disease activity index; QoL = quality of life; CYP = children and young people.

The interventions were either daily BC or a comparator milk. As a natural product, the composition of BC varies but is typically composed of 20% fat, 18% lactose and 55% protein (16% immunoglobulin) with energy value 460kCal/100g. A detailed subcomponent analyses of colostrum is provided in the review of BC constituents [12]. The placebo had a similar nutritional profile and was comprised of a mixture of milk powder (70%; Nestle Nido Instant Full Cream Milk Powder, Nestle) and milk protein concentrate (30%; Pure Milk Protein Concentrate 85, Bodybuilding Warehouse, Manchester, UK). Both products were supplied as powder by Colostrum UK Limited, London, UK (https://www.neovite.com/) and transported and stored at ambient temperature. The dose of both products was 20 g/day (4 rounded dessert spoons) made-up with about 150 mL of water, semi-skimmed or full cream milk or yoghurt. A range of flavourings (e.g., vanilla, chocolate), honey or sugar could be added to improve palatability. The milkshake could be taken at any time of day 2 hours after or 30 mins before eating and either in one go or split into two and stored in the refrigerator for up to 3 days. The dose could be reduced to 10g/day if CYP found the volume too great. In the subsequent open phase (7–12 weeks), all CYP received BC daily (Figure 1). The interventions were given alongside the young person’s usual diet but no other dietary supplements were provided. Children continued their usual treatment for CD and other conditions and there were no restrictions regarding use of medications.

To assess acceptability (primary outcome), CYP completed a daily symptom diary for 7 days at baseline and weeks 2, 6 and 12 (see supplementary methods). We also purposively sampled 20 CYP for qualitative interviews, conducted face to face or by telephone, undertaken by a member of staff independent of the clinical team (LB) at the end of weeks 1, 6 and 12 and with parents/carers after week 12 to explore the acceptability of the trial products, research methods and outcomes of the interventions (see supplementary methods). The interviews were semi-structured, guided by a topic sheet and audio-recorded. We also assessed health-related quality of life (HRQOL) at the same timepoints using the IMPACT III questionnaire (see Appendix A) validated from age 8 years [15].

Disease status was assessed at baseline, 6 and 12 weeks by the wPCDAI [14], growth and laboratory assays. Laboratory variables included biomarkers of intestinal inflammation (faecal calprotectin), intestinal integrity (serum intestinal fatty-acid binding protein (iFABP) and endotoxin antibodies; stool α1–antitrypsin (AAT); urine lactulose/mannitol sugar permeability test), systemic inflammation (serum C-reactive protein (CRP); serum α-1 acid glycoprotein (AGP); erythrocyte sedimentation rate (ESR), platelets, serum multiplex cytokine assay) and growth factors (serum insulin-like growth factor-1 (IGF-1), IGF binding protein 3 (IGF BP3)). See supplementary material for details of sample collection and laboratory methods. Where possible, research visits coincided with routine clinical care (e.g., out-patient appointments; infliximab infusions). Serious adverse events occurring from the start to 7 days after trial treatment were recorded. We planned to recruit 50 children in this feasibility study.

### 2.3. Statistical Methods

Clinical, biomarker and quality of life outcomes were analysed using standard statistical approaches for parametric and non-parametric data. To assess the effects of the intervention we compared the change from baseline in parameters over the first 1–6 weeks (blinded phase) between BC and placebo. We also report change over 1–12 weeks with those occurring over 1–6 weeks in children allocated to the BC arm and change from baseline in all CYP who took BC for 6 weeks (i.e., weeks 1–6 in CYP allocated to BC and weeks 7–12 in those initially allocated to placebo but taking BC during the open phase).

We analysed the interviews by coding the qualitative data according to a Framework analysis approach under the themes of acceptability of the trial products, research methods and outcomes of the interventions. Analysis was conducted by two members of the team (LB, BC). Further details regarding the qualitative methods and symptom diaries are shown in supplementary materials.

## 3. Results

We screened 247 CYP on 386 occasions and recruited 23 out of our original target of 50 patients (Figure S1: Trial profile). The study target was revised to 25 in a protocol amendment. The overriding reason for the low recruitment was failure to meet our restrictive inclusion criteria (n = 251 occasions) which included other diagnosis or CD not confirmed (78), already receiving a nutritional supplement (64), clinically unstable or recent change in treatment (61) and outside of age range or transitioned/being transitioned to adult care (27). Amongst 66 CYP who declined to participate, 24 either gave no reason or felt unable to comply with the study requirements, and 18 were currently stable and unwilling to change their management. On only 31 (8.0%) occasions did the CYP fail to participate because they were allergic, intolerant or disliked cow’s milk or nutritional supplements (Figure S1). Another issue that impacted on recruitment was that the study was suspended from 16 March to 23 July 2020 due to the COVID-19 restrictions and we perceived understandable reluctance amongst patients to attend the hospital after study re-start. Strategies adopted to increase recruitment are listed in the supplementary materials. 

The median (inter-quartile range; IQR) age was 15.2 (13.9–16.1) years, 9 (39.1%) were girls and 20 (87.0%) were white British. The median (IQR) duration of CD was 3.3 (1.2–4.6) years. Most CYP had ileocolonic, non-stricturing, non-penetrating CD and about two-thirds were in clinical remission. Eight (34.8%) CYP had peri-anal disease. Fatigue (8/21; 38.1%), abdominal pain 6/21; 28.6%) and joint pains (6/21; 28.6%) were the most common extra-intestinal disease manifestations. 12/21 (57.1%) CYP were receiving an immune modulator and 16/21 (76.2%) a biological/biosimilar. Demographic and clinical variables at recruitment were broadly similar in the two study arms; all CYP were either in remission or had mild disease (Table 1). 

### 3.1. Adherence with Trial Procedures and Milk Products

In the blinded phase over weeks 1–6, a similar proportion of CYP in the BC and placebo arms completed follow-up (8/12, 75.0% and 9/11, 81.8% respectively; *p* = 0.64). In the diaries completed during week 2, a similar proportion of CYP in the BC and placebo arms reported that they took all the milk product every day (5/7, 71.4% and 6/7, 85.7% respectively; *p* = 1.0; Table S1). In the week 6 diaries, optimal adherence (5/8; 62.5% in both arms), feeling well each day after drinking the milk (*p* = 1.0) and feeling better at the end of the week were similar in both arms (*p* = 0.56; Table S1).

In the 7–12 week open phase when all CYP received BC, 12 (70.6%) completed follow-up. Withdrawals tended to be less common in CYP who continued on BC from week 6 (1/8; 12.5%) than those who had switched from placebo milk to BC (4/9; 44.4%; *p* = 0.29). In the former group, 7/12 (58.3%) CYP tolerated BC over a total of 3 months. Whilst taking BC, minor adverse events contributed to the withdrawal of 3 CYP and an unrelated serious adverse event in one child (Figure S1).

Eleven CYP completed week 12 diaries with 10 providing information for all 7 days and 1 for 6 days only. Median (IQR, range) adherence score was 1.0 (0.86–1.0; 0.50–1.0) and 6 (54.5%) CYP took all the bovine colostrum on every day of the week. When asked how they felt about staying on the milk, 5 (45.5%) responded “that would be no problem”, 1 (9.1%) was “OK about it” but 4 (36.4%) reported “do not want to take it any longer”.

### 3.2. Clinical Outcomes

Average wPCDAI score fell during the course of study to a similar degree in the intervention and control arms (Table 2). Gains in weight and height and changes in quality-of-life scores were also similar in the two study arms. At the review at 6 weeks, 1/8 (12.5%) CYP receiving BC had worsened (transitioned from mild to moderate disease) whilst in those receiving placebo, 3/9 (33.3%) had worsened (from remission to mild) whilst 2/9 (22.2%) had improved (mild to remission). Change in disease activity, height and weight gain and quality of life were similar over 1–12 weeks in CYP who stayed on BC as over 1–6 weeks (Table 2). In the open phase (7–12 weeks; all CYP receiving BC), 3/11 (27.3%) improved (mild to remission) and none worsened. 

**Figure 2:**
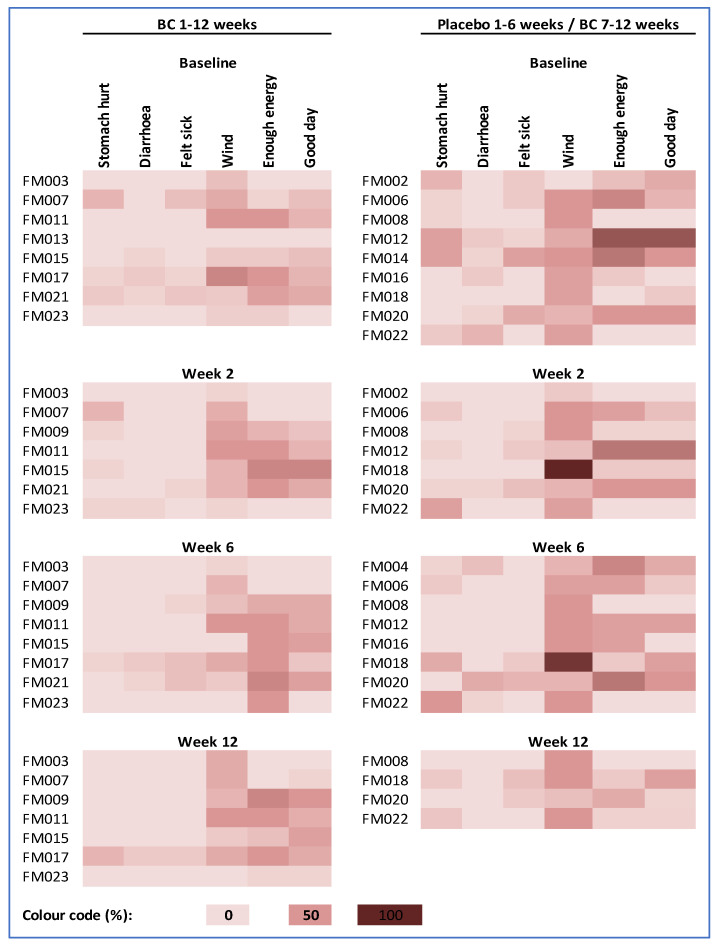
Average 7-day symptom and well-being scores for individual participants. Colour scale ranges from 0% (an absence of all four symptoms, lots of energy and had a good day on every day of the week) to 100% (the highest symptom score, no energy and an awful day on every day of the week).

Symptoms and well-being recorded by seven-day diaries at recruitment and during weeks 2, 6 and 12 were broadly similar in the two groups (Figure 2). Wind and lacking energy were the most commonly reported symptoms, and these tended to persist in both study arms. The lack of diary data for weeks 7–12 in the placebo group reflects the greater withdrawals in children who switched from placebo to BC. 

### 3.3. Laboratory Outcomes

Routine haematology and biochemical indices (Table S2) and concentrations of biomarkers of intestinal inflammation (faecal calprotectin), mucosal integrity in stools (faecal AAT), plasma (IFABP, anti-IgA, IgG and IgM endotoxin) and urine (lactulose/rhamnose ratio), systemic inflammation (plasma CRP and AGP) and growth factors (plasma IGF-1 and IGFBP3; Table 3) were broadly similar in the two arms at baseline and fell in the normal range, consistent with their remission/mild disease status. In the small number of participants, no clear differences were seen in routine indices or biomarker concentrations between the two arms during the initial 1–6 weeks, in CYP who took BC for 12 weeks or in all children after taking BC for 6 weeks (Table 3 and Table S2). Due to technical difficulties with the multiplex PCR assay results were generated from only a small number of participants (Table S3). 

### 3.4. Qualitative Findings

Qualitative interviews conducted with 14 CYP (9 BC and 5 placebo) and 14 parents (34 interviews altogether across the three timepoints) revealed that most had taken nutritional supplements previously and found a daily supplement acceptable. Overall, opinions were divided as to whether CYP preferred BC to the placebo milk and to other nutritional supplements. CYP and families reported devising their own ways of preparing the BC and flavouring to improve acceptability. Overall, the research methods were perceived as acceptable, although two CYP expressed a dislike of providing blood samples and one stool samples. See supplementary file for a more detailed report of the qualitative findings including factors related to participating in the study, acceptability of the research methods, and experiences in making up and taking the milk supplements.

## 4. Discussion

BC was acceptable as a daily nutritional supplement alongside their normal diet to most CYP and many were able to sustain BC daily for 12 weeks. 

Median time to clinical relapse in 109 children responding to EEN in Scotland was 6.5 (inter-quartile range: 7) months [4]. Following remission after EEN in CYP in Germany, 32/48 (67%) children relapsed during 12-months follow-up [7]. In a cross-sectional survey of CYP in The Netherlands, 28/82 (34%) had active disease and 20/54 (37%) in clinical remission had persistent intestinal inflammation [6]. Despite the effectiveness of EEN in the initial management of CD [3], and that further research into diet in the management of mildly active or inactive inflammatory bowel disease is a priority amongst patient and clinical representatives [16], few studies have explored long-term use of nutritional supplements for reducing disease severity and preventing disease flares. In two retrospective studies, supplementary feeds alongside usual diet reduced relapses, maintained clinical remission and improved growth [17,18]. Continued nutritional therapy with specific dietary restrictions may also prevent clinical relapse and reduce intestinal inflammation [19]. 

Although partial enteral nutrition is more acceptable to children than EEN [20], a major problem with nutritional therapy is the poor tolerance of FSMPs which often require a naso-gastric tube for administration [8,11]. In a retrospective study in Scotland, following EEN, only 15/48 (31%) children were able to continue feeds once normal diet was re-introduced [18]. In a survey of 29 children who had been managed with EEN and their parents/carers, the majority expressed a preference for alternative novel, solid food-based diets rather than further EEN [21].

BC contains 100-fold more immunoglobulins than human milk and high concentrations of the antimicrobial peptides lactoferrin, lactoperoxidases, lysozyme and oligosaccharides. Immunoregulatory cytokines include transforming growth factor-α, important in maintaining epithelial function and integrity, and high concentrations of transforming growth factor-β which has anti-inflammatory effects, regulates cellular proliferation, differentiation and repair and is essential in the induction of regulatory T cells. Additional growth-promoting and mucosal repair factors include insulin-like growth factors 1 and 2, vascular endothelial factor, basic fibroblast growth factors and platelet-derived growth factor [12]. Improvements in processing conditions results in extended shelf-life of BC-based products with minimum loss of bioactive components [22]. 

Supported by findings in animal models of intestinal inflammation, BC may provide therapeutic benefits in a range of gastrointestinal diseases including inflammatory bowel disease although clinical studies are few [13]. In a case series of 6 children with moderate-to-severe CD managed with an exclusion diet and nutraceutical therapy that included BC, all achieved clinical remission, a fall in systemic inflammatory markers and improved quality of life within 2 months. Clinical remission was sustained between 18 and 90 months and no significant adverse effects were reported [23]. BC enemas in adults with distal colitis improved symptom and histological scores [24]. The small number of children recruited to our trial, their mild/moderate disease status, continuation of other treatments for CD including infliximab in most children and the limited intervention period prevented the assessment of the potential of BC to improve clinical and laboratory outcomes. There is insufficient research evidence at present to undertake a systematic review of the effectiveness of BC in CD and we are not aware of any on-going trials in children or adults with CD. 

In a systematic review of clinical trials of gastrointestinal and other diseases and exercise tolerance/athletic performance, BC was generally well tolerated, and no serious side effects were reported in the 2326 participants. Some people reported mild adverse effects including an unpleasant taste, nausea, flatulence, diarrhoea, skin rash, and unspecified abdominal discomfort [13]. Daily BC was also well tolerated for up to 12 weeks in our study especially amongst children who continued on BC throughout the 12-week study period. Given that the comparator in our study was milk powder rather than a FSMP, the acceptability and findings from the qualitative research suggest that BC may be more acceptable for long-term use than current FSMPs.

The BC used in the current study was packaged from a commercially available powdered form and was stable for at least 12 months in a cool room environment. Although other forms of processed BC are potentially available, caution is needed as bioactivity of BC is highly sensitive to heat exposure [25]. BC-containing consumer products that have undergone a heating/baking stage have a significant risk of loss of bioactivity (such as pro-reparative growth-factor activity or, in the case of IgG, antigen binding), even though the apparent concentration of IgG or individual growth-factor level appear unchanged [26]. Although CYP continued their usual diet during the study, a concern is the potential adverse effects of additives to improve the palatability of BC. These include high fructose containing foods, such as honey, as a high fructose diet had a pro-colitic effect in a mouse model of inflammatory bowel disease [27]. This should be considered in future trials.

A strength of our study was the use of a range of methods to assess the acceptability and efficacy of BC using a combination of clinical and laboratory outcomes, patient diaries and qualitative research. These research methods were well accepted and can inform the design of future clinical trials. A major limitation of our study was the very low recruitment rate (23/247; 9.3%) despite the screening of a large number of CYP. Although this was likely partly due to the limitations resulting from the COVID-19 pandemic, subsequent trials could have less restrictive inclusion and exclusion criteria. Although the small number of CYP recruited impaired our ability to assess the efficacy of BC, we report our clinical and laboratory outcomes to inform the design of subsequent trials.

## 5. Conclusions

The known biological properties of BC, the evidence of benefit in animal models of intestinal inflammation and limited human trials and its acceptability in this study suggest that a larger trial evaluating its role in long-term management in CYP also taking their usual diet is indicated. Given the frequent use of dietary supplements in our study, a comparison of BC against a well-established FSMP and with less restrictive inclusion/exclusion criteria would be appropriate. 

## Figures and Tables

**Table 1:** Demographic and clinical variables at recruitment according to intervention arm.

	First Milk N = 12	Placebo N = 11
**Demographic variables**		
Age (median, IQR; months)	15.4 (13.4–16.4)	14.9 (13.9–16.0)
Female (No; %)	5 (41.7)	4 (36.4)
Ethnicity White British (No; %)	11 (91.7%)	9 (81.8%)
**Clinical Variables**		
Age at diagnosis (median, IQR; years)	12.3 (8.9–14.4)	11.9 (9.6–14.3)
Height ^1^ (median, IQR; m)	161.8 (149.8–170.4)	164.5 (155.8–171.9)
Weight ^1^ (median, IQR; kg)	57.4 (49.6–61.6)	53.2 (43.0–63.2)
Disease location ^2^ (No; %)		
L1: distal 1/3 ileum ± limited caecal disease	0 (0.0)	1 (9.1)
L2: colonic	2 (16.7)	3 (27.3)
L3: ileocolonic	7 (58.3)	7 (63.3)
L4a: upper disease proximal-Ligament of Treitz	0 (0.0)	1 (9.1)
L4b: upper disease distal-ligament of Treitz and proximal-distal 1/3 ileum	2 (16.7)	2 (18.2)
Disease behaviour (No; %)		
B1: non-stricturing; non-penetrating	9 (66.7)	9 (81.8)
B2: structuring	0 (0.0)	1 (9.1)
B2B3: both penetrating and stricturing disease, either at the same or different times	1 (8.3)	0 (0.0)
p: perianal disease modifier	5 (41.7)	3 (27.3)
Growth delay (height for age Z score < −1.64) (No; %)	0 (0.0)	1 (9.1)
Disease activity ^3^ (No; %)		
<12.5–remission	8 (72.7)	7 (70.0)
12.5–40.0–mild	3 (30.0)	3 (27.3)
wPCDAI score (median; IQR)	0.0 (0.0–25.0)	7.5 (5.0–30.0)
Extra-intestinal manifestations ^4^ (No; %)		
joint pain	4 (40.0)	2 (18.2)
abdominal pain	3 (30.0)	3 (27.3)
erythema nodosum	1 (10.0)	0 (0.0)
mouth ulcers	2 (20.0)	2 (18.2)
iritis	0 (0.0)	0 (0.0)
fatigue	4 (40.0)	4 (36.4)
fistula	1 (10.0)	1 (9.1)
fissure	1 (10.0)	0 (0.0)
Current treatment ^5^ (No; %)		
oral/rectal aminosalicylate	1 (10.0)	1 (9.1)
immune modulator	5 (50.0)	7 (63.6)
biological or biosimilar	7 (70.0)	9 (81.8)
Past medical history (No; %)		
oro-facial granulomatosis	3 (25.0)	3 (27.3)
iron deficiency	1 (8.3)	0 (0.0)
erythema nodosum	1 (8.3)	0 (0.0)
asthma/eczema	0 (0.0)	1 (9.1)
candida oesophagitis	0 (0.0)	1 (9.1)
nocturnal enuresis	0 (0.0)	1 (9.1)
short stature/delayed puberty	0 (0.0)	1 (9.1)
Quality of life ^6^ (No.; median; IQR)	63.0 (49.0–82.0)	63.5 (59.0–74.0)

Notes: ^1^. Height and weight available for 10 CYP allocated to BC and 10 controls; ^2^. Some children had disease in more than one location; ^3^. Weighted paediatric Crohn’s disease activity index available in 11 CYP allocated to BC and 10 controls; ^4^. Data available for 10 CYP allocated to BC and 11 controls; ^5^. Data available for 10 CYP allocated to BC and 11 controls; none were receiving steroids; ^6^. IMPACT III total score; available in 7 CYP allocated to BC and 10 controls.

**Table 2:** Clinical variables at recruitment and change according to intervention arm.

Clinical Variable	Change Weeks 1–6 No. Median (IQR)		*p* Value	Change Weeks 1–12 No. Median (IQR)	Change in All Children after 6 Weeks BC No. Median (IQR)
	BC	Placebo		BC	BC
Disease actvity ^1^	7 −10 (−15.0–5.0)	10 −1.25 (−13.1–13.1)	1.0	4 −15.0 (−28.8–−1.25)	12 −2.5 (−13.75–4.38)
Height (cm)	9 0.7 (−0.45–1.6)	10 1.0 (0.075–2.4)	0.07	8 0.7 (−0.13–1.68)	13 0.5 (−0.55–1.55)
Weight (kg)	9 1.2 (−0.5–3.2)	10 1.6 (−0.74–2.48)	0.37	8 1.55 (−1.24–2.98)	13 0.5 (−0.55–1.5)
Quality of life ^2^	6 −1.5 (−10.0–1.0)	9 −12.0 (−15.0–−4.0)	0.15	4 −12.5 (−17.8–−2.8)	11 0.0 (−7.0–4.0)

^1^ Weighted paediatric Crohn’s disease activity index. ^2^ IMPACT III total score.

**Table 3:** Laboratory biomarkers according to intervention arm.

Biomarker/Variable	Baseline No. Median (IQR)		Change Weeks 1–6 No. Median (IQR)		*p* Value	Change Weeks 1–12; FM Group No. Median (IQR)	Change in All Children after 6 Weeks FM No. Median (IQR)
	First Milk	Placebo	First Milk	Placebo			
Intestinal inflammation							
Faecal calprotectin (μg/g stool) Normal <50 μg/g stool	8 23.5 (14.5 to 60.3)	7 80.0 (10.0 to 532.0)	7 −9.0 (−14.0 to 61.0)	6 −45.5 (−322.8 to −0.5)	0.59	7 −1.0 (−14.1 to 724.0)	12 −10.05 (−35.5 to 2.25)
Mucosal integrity							
Faecal alpha 1–antitrypsin (mg/dl) Normal <26.8 mg/dl	7 31.2 (11.7 to 40.4)	8 13.4 (7.7–175.3)	6 −11.9 (−29.0 to 1.74)	7 5.11 (0.00 to 10.1)	0.10	6 −17.3 (−31.4 to −4.13)	11 −2.86 (−20.9 to 12.6)
Plasma intestinal fatty acid binding protein (pg/mL) Normal range 230–1800 pg/mL	11 746.0 (352.4 to 1494.5)	10 521.0 (360.3 to 695.8)	8 −257.6 (−633.3 to 115.6)	9 94.2 (−21.0 to 245.1)	0.15	8 −23.9 (−473.0 to 515.5)	13 75.9 (−329.6 to 261.5)
Plasma anti-IgA endotoxin (AMU/mL)	11 50.6 (31.2 to 70.1)	10 25.8 (19.3 to 74.4)	8 −0.2 (−15.8 to 4.1)	9 −1.24 (−11.3 to 4.02)	1.0	8 0.93 (−9.87 to 18.8)	13 −0.46 (−12.7 to 3.89)
Plasma anti-IgG endotoxin (GMU/mL)	11 118.3 (64.7 to 127.7)	10 53.6 (29.6 to 138.8)	8 35.3 (−56.6 to 99.7)	9 −6.3 (−56.6 to 40.0)	0.35	8 −10.8 (−22.3 to 29.1)	13 14.6 (−26.0 to 91.1)
Plasma anti-IgM endotoxin (MMU/mL)	11 110.8 (86.3 to 161.4)	10 65.0 (48.9 to 115.7)	8 −0.48 (−20.5 to 42.6)	9 1.3 (−3.67 to 16.4)	1.0	8 5.55 (−19.7 to 25.2)	13 −1.97 (−18.6 to 17.9)
Urine sugar permeability test (ratio of % recovery lactulose/rhamnose)	10 0.030 (0.021 to 0.044)	10 0.030 (0.026 to 0.045)	8 −0.0046 (−0.0064 to 0.012)	9 −0.0046 (−0.014 to 0.0047)	1.0	8 0.0021 (−0.020 to 0.015)	13 0.0033 (−0.0059 to 0.011)
Systemic inflammation							
Plasma C-reactive protein (mg/L) Normal range 0.11–4.52 mg/L	11 3.90 (3.90 to 4.00)	10 4.00 (3.90 to 4.73)	8 0.0 (0.0 to 0.08)	9 0.0 (0.0 to 0.0)	0.21	8 0.0 (−1.0 to 0.0)	13 0.0 (−0.05 to 0.0)
Plasma alpha 1 acid glycoprotein (μg/mL) Normal range 286–1087 μg/ml	11 1002.0 (599.2 to 1501.5)	10 814.2 (575.1 to 967.4)	8 78.2 (−66.3 to 230.9)	9 42.0 (−369.4 to 376.9)	1.0	8 −6.4 (−220.6 to 253.1)	13 −93.6 (−937.3 to 109.4)
Growth factors							
Insulin-like growth factor−1 (ng/mL) Normal range 43–182 ng/ml	11 244.2 (169.5 to 312.8)	10 264.6 (237.6 to 283.6)	8 9.95 (−68.1 to 48.6)	9 −3.77 (−27.3 to 27.7)	1.0	8 9.3 (−55.0 to 19.9)	13 38.0 (−36.4 to 47.72)
Insulin-like growth factor binding protein−3 (ng/mL) Normal range 1553–3089 ng/ml	11 2550.4 (2238.4 to 3017.1)	10 2624.6 (2506.6 to 3104.9)	8 −9.09 (−42.6 to 236.9)	9 144.4 (−252.0 to 284.4)	0.153	8 74.7 (−338.7 to 497.5)	13 39.96 (−37.1 to 507.2)

## Data Availability

Applications for access to study data will be reviewed by the study investigators and permission granted for reasonable requests.

## Supplementary Materials

The following supporting information can be downloaded at: https://www.mdpi.com/article/10.3390/nu14214598/s1. Figure S1: Trial profile. Table S1: Diaries completed during the blinded phase (weeks 1–6). Table S2: Routine haematology and biochemistry according to intervention group. Table S3: Multiplex immunoassay results according to intervention and follow-up. References [28,29,30] are cited in the supplementary materials.

## Trial registration

International Standard Randomised Controlled Trials Number (ISRCTN) Register: 78570360. Registered 1 July 2019, https://doi.org/10.1186/ISRCTN78570360.

## Appendix A. Impact–III Questionnaire (UK)

### Instructions

Below you will find a questionnaire containing 35 questions for children who have inflammatory bowel disease (Crohn’s disease or ulcerative colitis). The questions are about your life with inflammatory bowel disease. Some questions deal with, for example, pains you may suffer from, others are about feelings or worries you may have.

After each question you will see boxes above five possible answers. Please put a cross in the box above the answer that best fits your answer.

First, an example.

**Question 1.**

**How much has your stomach been hurting you in the past two weeks?**

☐ Not at all	☐ A little	☐ Quite a bit	☐ Much	☐ Very much

**Question 2.**

**Taking medicines or tablets bothers you.**

☐ Not at all	☐ A little	☐ Quite a bit	☐ Much	☐ Very much

**Question 3.**

**Has your inflammatory bowel disease prevented you from eating what you want in the past two weeks?**

☐ Not at all	☐ A little	☐ Quite a bit	☐ Much	☐ Very much

**Question 4.**

**How much have you been worrying about having a flare-up (increase of symptoms) in the past two weeks?**

☐ Not at all	☐ A little	☐ Quite a bit	☐ Much	☐ Very much

**Question 5.**

**How much does it bother you that you have an illness that does not just go away?**

☐ Not at all	☐ A little	☐ Quite a bit	☐ Much	☐ Very much

**Question 6.**

**How much energy did you have during the past two weeks?**

☐ Very much energy	☐ Much energy	☐ Quite a bit of energy	☐ A little energy	☐ No energy at all

**Question 7.**

**How do you feel about your weight?**

☐ I feel great about my weight	☐ I feel good about my weight	☐ I don’t feel good or bad about my weight	☐ I feel bad about my weight	☐ I feel awful about my weight	

**Question 8.**

**How has your inflammatory bowel disease affected your family?**

☐ The effect has been great	☐ The effect has been good	☐ It has not affected our family	☐ The effect has been bad	☐ The effect has been awful

**Question 9.**

**How often did you have to miss out on certain things (hobbies, play, parties) because of your inflammatory bowel disease in the past two years?**

☐ Never	☐ Rarely	☐ Sometimes	☐ Often	☐ Very often

**Question 10.**

**How often have you been bothered by diarrhoea (loose or frequent bowel movements) in the past two weeks?**

☐ Never	☐ Rarely	☐ Sometimes	☐ Often	☐ Very often

**Question 11.**

**How much do you worry about health problems you might have in the future?**

☐ Not at all	☐ A little	☐ Quite a bit	☐ Much	☐ Very much

**Question 12.**

**How often do you think it is unfair that you have inflammatory bowel disease?**

☐ Never	☐ Rarely	☐ Sometimes	☐ Often	☐ Very often

**Question 13.**

**During the past two weeks, were you ever angry that you have inflammatory bowel disease?**

☐ Never	☐ Rarely	☐ Sometimes	☐ Often	☐ Very often

**Question 14.**

**Do you think too many rules or limits are placed on you because of your inflammatory bowel disease?**

☐ Not at all	☐ A little	☐ Quite a bit	☐ Much	☐ Very much

**Question 15.**

**How do you feel about the way you look?**

**☐** **I think I look** **great**	**☐** **I think I look** **good**	**☐** **I don’t think I** **look good or** **bad**	**☐** **I think I look** **bad**	**☐** **I think I look** **awful**

**Question 16.**

**Are you embarrassed because of your bowel condition?**

☐ Not at all	☐ A little	☐ Quite a bit	☐ Much	☐ Very much

**Question 17.**

**Did you have fun during the past two weeks?**

☐ Not at all	☐ A little	☐ Quite a bit	☐ Much	☐ Very much

**Question 18.**

**Is it harder to make friends because of your inflammatory bowel disease?**

☐ Not at all	☐ A little	☐ Quite a bit	☐ Much	☐ Very much

**Question 19.**

**How often do you worry about your stool (bowel movement) containing blood?**

☐ Never	☐ Rarely	☐ Sometimes	☐ Often	☐ Very often

**Question 20.**

**Are you worried you cannot have a boyfriend or girlfriend because of your inflammatory bowel disease?**

☐ Not at all	☐ A little	☐ Quite a bit	☐ Much	☐ Very much

**Question 21.**

**How often did you feel sick in the past two weeks?**

☐ Never	☐ Rarely	☐ Sometimes	☐ Often	☐ Very often

**Question 22.**

**How do you feel about the tests you have to go through?**

☐ I do not mind them at all	☐ I mind them a tiny bit	☐ I mind them a little	☐ I mind them a lot	☐ I hate them

**Question 23.**

**Do other children bully you or leave you out of things because of your inflammatory bowel disease or its treatment?**

☐ Never	☐ Rarely	☐ Sometimes	☐ Often	☐ Very often

**Question 24.**

**How often do you worry about having an operation?**

☐ Never	☐ Rarely	☐ Sometimes	☐ Often	☐ Very often

**Question 25.**

**How often are you afraid you might have an accident (not get to the toilet in time)?**

☐ Never	☐ Rarely	☐ Sometimes	☐ Often	☐ Very often

**Question 26.**

**Do you try to hide your inflammatory bowel disease?**

☐ No, I do not try at all	☐ I don’t try much	☐ I try a little	☐ I try hard	☐ Yes, I try very hard

**Question 27.**

**Does your inflammatory bowel disease make it difficult to travel or go on holiday?**

☐ No, not difficult	☐ Slightly difficult	☐ Quite difficult	☐ Very difficult	☐ Yes, extremely difficult

**Question 28.**

**How did you feel during the past two weeks?**

☐ Great	☐ Good	☐ Not good or bad	☐ Bad	☐ Awful

**Question 29.**

**Are you happy with your life?**

☐ Yes, very happy	☐ Happy	☐ Not happy or unhappy	☐ Unhappy	☐ Very unhappy

**Question 30.**

**Do you feel there is someone you can talk to about your inflammatory bowel disease?**

☐ Always	☐ Often	☐ Sometimes	☐ Rarely	☐ Never

**Question 31.**

**How often did you have to pass wind in the past two weeks?**

☐ Never	☐ Rarely	☐ Sometimes	☐ Often	☐ Very often

**Question 32.**

**How tired have you felt in the past two weeks?**

☐ Not at all tired	☐ A little tired	☐ Quite tired	☐ Tired	☐ Very tired

**Question 33.**

**How do you feel about your height?**

☐ I feel great about my height	☐ I feel good about my height	☐ I don’t feel good or bad about my height	☐ I feel bad about my height	☐ I feel awful about my height

**Question 34.**

**Despite your inflammatory bowel disease, can you take part in as much sport as you would like?**

☒ Yes, as much as I would like	☐ Almost as much as I would like	☐ About half as much as I would like	☐ Less than half as much as I would like	☐ Much less than I would like

**Question 35.**

**How often are you able to go to school?**

☐ Always	☐ Often	☐ Sometimes	☐ Rarely	☐ Never
