# Supplementary material for: Dietary Therapy to Improve Nutrition and Gut Health in Paediatric Crohn’s Disease; A Feasibility Study
Source: Nutrients. 2022 Nov 1;14(21):4598. doi: 10.3390/nu14214598 (PMC9655551; doi:10.3390/nu14214598)
Supplement: Supplementary file 1 [file nutrients-14-04598-s001.zip › Allen SJ et al_supplementary materials.pdf]

Dietary therapy to improve nutrition and gut health in paediatric Crohn's disease; a feasibility study

Supplementary materials

Supplementary methods

a) Symptom and compliance diaries

All CYP supported by their parents/carers, as appropriate, were asked to keep daily diaries for one week before starting the intervention and at weeks 2, 6 and 12. The format and content of the diary was informed by the activities-based PPI workshop. The diary recorded information on symptoms, adherence and responses to/experiences of tests/procedures and used a mix of closed, scaling questions (e.g., non-smiley-smiley faces) and some open questions to elicit key data.

Symptoms for each day (stomach hurt, diarrhoea, felt sick, wind) and well-being (enough energy, whether it was a good day) were scored as either 0 (symptom absent/lots of energy/good day), 1 (moderate symptoms/a bit of energy/OK day) or 2 (severe symptom/no energy/awful day). An average for each score over the 7 days was calculated and expressed as a percentage of the total possible score (14). Therefore, 0 (0%) would be an absence of all four symptoms, lots of energy and had a good day on every day of the week whereas 14 (100%) would be the highest symptom score, no energy and an awful day on every day of the week. These data were then illustrated in a heat map (see Figure 2) using conditional formatting in MS Excel.

In the diaries for weeks 2, 6 and 12, CYP reported how much of the milk they had taken each day ("I took all of it", "I took some of it" or "I took none of it") and in the diaries for weeks 6 and 12, how they felt after taking the milk ("I felt really good", "I felt OK" or "I felt awful") and both parameters were scored as 1, 0.5 or 0 respectively. These scores were averaged for the week based on how many days the diary was completed ("adherence score").

At the end of week 6 CYP were asked "Overall, do you think the milkshake has made you feel better?" and responded "Definitely felt better on the milkshake", "No difference" or "Felt worse on the milkshake". In the week 12 diary, CYP were asked "How would you feel about staying on the milkshake every day?" and responded "That would be no problem",

“OK about it” or “Do not want to take it any longer”. The front cover of the baseline diary is shown below. Diaries are available on request.

“First milk” in paediatric Crohn’s disease study

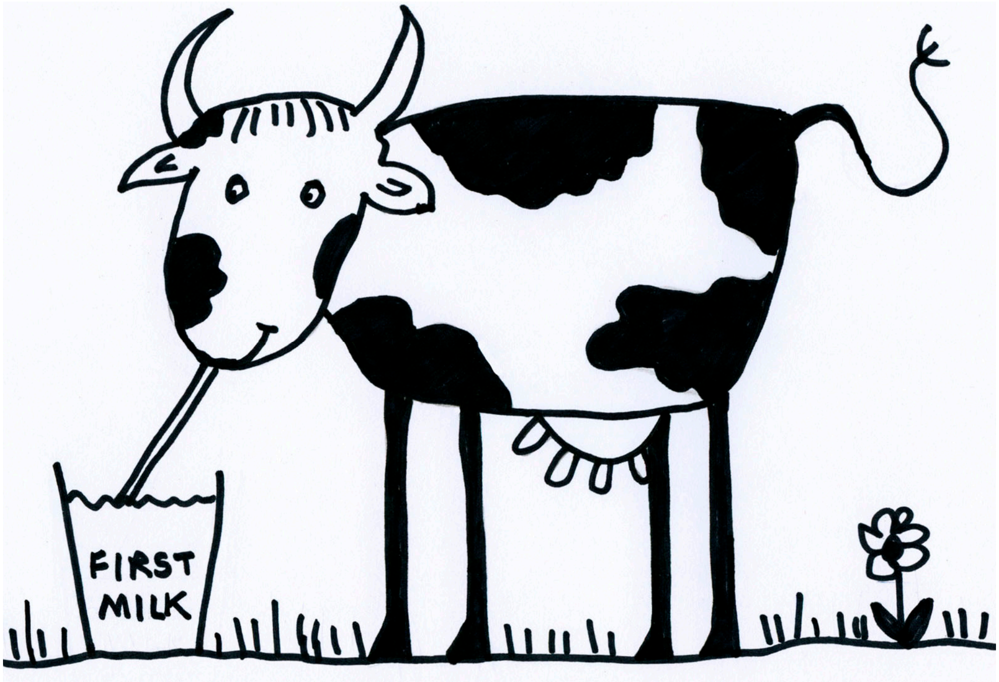

Daily Diary for Week before starting milkshake

Study ID:
Date handed to child/young person:
Please return your diary: (details of how and to whom)
If you have any questions about the diary, please contact: (details to be inserted here)

First Milk in Paediatric Crohn’s disease study; week 0 diary
v1.0 13 November 2018 Study ID:

b) Qualitative methods

In selecting CYP for interview, we liaised with the TCTU to ensure that each study arm was represented without unblinding. Interviews were conducted face-to-face in the home setting (n=2) or by telephone (n=32) by LB. When interviewing the CYP, their parent/carer could be present if the CYP or parent/carer felt this was appropriate. With the permission of CYP and parents, interviews were audio-recorded. Interview guides are shown below.

The qualitative data was analysed systematically using Framework Analysis[1,2] using a staged approach: transcription; familiarisation with the interview; coding; developing a working analytical framework; applying the analytical framework; charting the data into the framework matrix and interpreting the data. Codes and categories were developed and indexed, analytic memos resulted in the development of key themes (interpretive concepts) generated through comparison between and within cases (CYP/families). The analysis was undertaken by two experienced qualitative researchers (LB, BC) with differences in interpretation explored and subjected to critical dialogue and reflexive consideration until a consensus was reached. We liaised with members of the PPI Advisory Group and the young person with CD from the GenerationR Young Persons' Advisory Group to explore the preliminary findings and ensure that these were grounded in the reality of the lives of children and their parents.

Indicative topic guide for interviews with children and young people

Purpose of the interviews

The study protocol noted that interviews would focus on two key issues of interest to the feasibility trial:

- Factors relating to adherence to the dietary therapy, collection of samples and data collection (e.g., what were children's experiences of making up and having to drink the milk, what were the barriers, what helped, what could improve adherence in a future trial; satisfaction with the research methods and materials)
- Factors relating to outcomes of dietary therapy (e.g., what outcomes are important to the young person and parent/carer; are these being adequately addressed within the current framework; how could these be measured/assessed).

---

[1] Ritchie J, Spencer L 1994. "Qualitative data analysis for applied policy research" in A. Bryman and R. G. Burgess [eds.] "Analyzing qualitative data", 1994, pp.173-194. London: Routledge.

[2] Gale NK, Heath G, Cameron E, Rashid S, Redwood S. Using the framework method for the analysis of qualitative data in multi-disciplinary health research. BMC Medical Research Methodology 2013;13:117.

Obtaining informed consent and assent

The researcher introduced themselves and reviewed the CYP's understanding of the study. The researcher provided the opportunity for the CYP and/or parent to ask questions and/or clarify their involvement and confirm that they still assented/consented to participate (parent consent for child <16yrs was checked prior to talking to the child/young person).

The CYP was made aware at the start of the interview that the interview will be audio taped and asked if this was okay. If they were not happy for this, then notes were taken. The CYP was made aware that the interview could be stopped at any time and that there were no right or wrong answers to the questions.

Interview style

The interview was as informal and conversational as possible to facilitate the CYP's comfort. Interviews were guided by the following topics and prompts but also followed-up on responses made by the CYP, as appropriate.

Interview guide

The key questions from this guide were used across the three interviews across. Each question is tagged to show when it was asked.

**Interview**	**Key question number**																				
	1	2	3	4	5	6	7	8	9	10	11	12	13	14	15	16	17	18	19	20	21
**1**	x	x	x	x	x	x	x			x	x										
**2**		x	x	x	x	x	x		x		x	x									x
**3**		x	x	x	x	x	x	x	x				x	x	x	x	x	x	x	x	x

Topic 1: About the milkshake

1. **BEFORE you started the study what did you think about drinking a milkshake every day? (Int 1 only)**
   Prompts: Did you think it would be OK? Did you wonder about what it would taste like?

2. **When do/did you drink the milkshake? (Int. 1 & 2 & 3)**
   Prompts: Do you drink it before school/after school/before bed? Is this a convenient time to drink it? Does it feel like a drink or medicine? Do you drink it with your medicines?

3. **Who makes/made the milkshake? Do you add any flavours or extra things to the milkshake? (Int. 1 & 2 & 3)**
   Prompts: What flavours? Which one is best? Do you/mum/dad/carer add sugar or honey?

4. **Now that you've been taking the milkshake for a week/6 weeks, what's it like to drink? (Int 1 & 2)**
   Prompts: Do you mind drinking it? Does someone have to nag you to drink it? What's good or bad about the milkshake? What does it taste like? Do you drink it all in one go?
   OR
   Now that you've finished taking the milkshake? (Int 3 only)
   Prompts: Did you mind drinking it? Did someone have to nag you to drink it? What was the best and worst things about the milkshake?

5. **Do/did you always finish all the milkshake? (Int. 1 & 2 & 3)**
   Prompts: If not, why not? (too big, don't like it, don't want to, rather be doing something else, no-one else has to take it, don't like medicine).

6. **What's it like after you've drunk it? (Int. 1 & 2 & 3)**
   Prompts: do you feel OK/full/sicky/yucky/burpy?

7. **What would you like to change/have changed about the milkshake (Int. 1 & 2 & 3)**
   Prompts: Would you like to change the amount, taste, after-taste, daily dose.

8. **Is there anything else you want us to know about the milkshake? (Int 3 only)**
   Prompts: Is there anything that worries you about taking the milkshake? Anything we should remember to tell other children about before they start on the study?

9. **Do you think that drinking the milkshake every day for 6 (maybe 12) weeks is too long or OK? (Int 2 & 3 only)**
   Prompts: How long do you think it is OK to have to drink the milkshake for?

Topic 2: About the study

10. **Did the information you were given about the study explain things well? Int 1 only)**
    Prompts: what else would you have liked to know? If you could have tasted the milkshake and seen the amount you had to drink before you did the study would this have helped you decide? Would you have said yes or no?

11. **Now you are taking the milkshake do you want any more information about it? ? (Int 1 & 2)**
    Prompts:

12. **Did/do you understand that the milkshake you have been drinking at the moment might not be the 'real' milkshake? (Int 2 only)**
    Prompts: How does that make you feel?

13. **Do you/did you mind filling in the diary? (Int 3 only)**
    Prompts: who fills in the diary – you/mum/dad? Is the diary a fun thing to fill in? what's good and bad about the diary?

14. **Did you ever feel like stopping being part of the study? (Int 3 only)**
    Prompts: When was this (start, middle, near the end, all the time) Why was this (fed up with taking milkshake, hated milkshake, got bored, didn't think it was working, felt poorly, no-one reminded me to take milkshake, didn't like not knowing if I was on the real milkshake)?

15. **What kept you motivated to keep on taking the milkshake and being part of the study (Int 3 only)**
    Prompts: Being part of a study, helping other children/making a difference, mum/dad/friends, wanting to see if it would help me get better.

16. **Do you/did you mind filling in the IMPACT III questionnaire? (need easier wording here) (Int 3 only)**
    Prompts: What's good and bad about filling in the questionnaire?

17. **Questions about the different samples (stool, urine and blood) for research purposes (Int 3 only)**
    Prompts:

18. **If we were to do a bigger study with lots more children and young people what do you think we should change to help make it a better experience for the children and young people?**
    Prompts: e.g., Shorter time to have to take milkshake? Better flavours? **(Int 3 only)**

Topic 3: Outcomes of importance to children and young people

19. **What do you think is the most important thing about your condition that you would like the milkshake drink to help with? (Int 3 only)**
    Prompts: Things like tummy ache, pain, bloating, diarrhoea… Things like feeling happy and well….Things like feeling poorly and tired…… Things like having to take medicines, having tests…… Things like being just like your friends.

20. **How do you think we should try and find out (measure/assess) whether children and young people think that the milkshake is working or not? (Int 3 only)**
    Prompts:

21. **Do you think that taking the milkshake has helped you? If so, how? (Int 2 & 3 only)**

Prompts:

Closing the interview

The CYP was asked if there is anything else they would like to add and then the interview was closed. The CYP and parent/carer, as appropriate, were thanked. It was ensured that the CYP had contact details of the researcher/research team if they had any questions or concerns.

Parent interviews (timepoint 3 only

c) **Sample collection**

At baseline and weeks 6 and 12, 5mls of venous blood and stool and urine samples were collected. CYP were provided with materials to collect a stool sample at home that was to be kept in the refrigerator either the evening or morning prior to a research visit.

Urine sugar permeability test

Participants were not tested if they had diabetes. Participants were requested to abstain from spicy foods in particular curries, alcohol and avoid non-steroidal anti-inflammatory drugs or aspirin for 5 days before taking the test.

Participants were provided with:

- Glass bottles containing Bottle 200mg mannitol, 1000mg lactulose and 1000mg Rhamnose refrigerated at 4°C until usage and supplied by Prof Ray Playford, University of West London. Dosage according to weight

Weight of Child	No of bottles	Dose	Total of tap water
14-24kg	2	800mg Mannitol<br>2000mg Lactulose<br>400 mg Rhamnose	140ml (70mls each bottle)
25-34kg	3	1200 mg Mannitol<br>3000mg Lactulose<br>600 mg Rhamnose	210ml (70mls each bottle)
≥35kg	4	1600mg Mannitol<br>4000mg Lactulose<br>800mg Rhamnose	280ml (70mls each bottle)

- 24 hour urine collection container containing Chlorhexidine (Tayside Pharmeuticals, Ninewells Hospital and Medical School, Ninewells, Dundee DD1 9SY)
- Measuring jug, pipette and small urine container
- Large plastic bags (Technical service consultant Ltd/www.tscswabs.co.uk)
- Female urinal for girls (Betterlife Healthcare.com)

Participants were provided with the following instructions:

- Eat a normal evening meal at least 4 hours before you are due to go to bed.
- Fast over the following 4 hours (no food or drink). Any urine produced during this period is not collected but flushed down the toilet.
- After 4 hours, a responsible adult adds ordinary tap water (between 140ml and 280ml depending on weight) to the contents of all the issued bottles which the CYP then drinks.
- Collect all urine produced for 8 hours after the test and the first urine passed on waking the next morning in the urine container.
- Continue to fast during this 8-hour period.
- Store the urine container at room temperature in a safe place.
- Record total volume of urine using the graduations on the container.
- Use the pipette to extract 14mls for the chlorhexidine solution tube.

On receipt at Alder Hey, the research nurses checked the total volume of urine collected from each participant, extracted 8mls (maximum) and separated the sample into 2 universal containers and stored them at -80°C until shipment to collaborators for the measurement of urinary sugars.

d) Laboratory analysis

Blood samples were divided equally into EDTA and lithium heparin anticoagulated tubes. The EDTA whole blood sample was used for the measurement of erythrocyte sedimentation rate (ESR) by the Westegren semi-micro method (Aquisel, Barcelona, Spain) and haemoglobin and mean cell volume using a Fluorocell PLT XN-2000 analyser (Sysmex, Milton Keynes, UK). The lithium heparin sample was centrifuged, the plasma removed and C-

reactive protein, urea, creatinine, calcium, phosphate, total protein, albumin, total bilirubin and liver enzymes were measured by photometery using Alinity ci-series autoanalysers (Abbot, Maidenhead, UK). The remaining plasma was transferred into screw cap microtubes and stored at -80°C until shipment to collaborators.

Plasma α_1-acid glycoprotein was measured by enzyme linked immunosorbent assay (ELISA; Rand D systems, Abingdon, UK) and plasma cytokines using a 20-plex immunoassay (ThermoFisher, UK). Plasma endotoxin antibodies (IgA, IgG and IgM), intestinal fatty acid binding protein (iFABP), insulin like growth factor (IGF)-1 and IGF binding protein 3 were measured by ELISA (Hycult, UK and R and D systems, Abingdon, UK). All assays were tested in duplicate in accordance with the manufacturers' guidelines. Quality controls were included with each batch of tests.

Faecal calprotectin was measured by ELISA on a Phadia 250 autoanalyser. The remaining stool samples were stored at -80°C until they were shipped to collaborators for measurement of α_1–antitrypsin by ELISA (IBL, Germany).

Urinary sugars

a. Urine analyses were performed in the Department of Chemical Pathology, Royal Cornwall Hospitals Trust, Truro under the supervision of Dr. Rachel Cooper. The testing and subsequent urine analyses was based on published methods.[3]
b. The system comprised of a Thermo-Scientific Dionex UltiMate 3000 UHPLC system using a Dionex CarboPac PA1 anion-exchange analytical column (4.5 X 250 mm) with an equivalent guard column. NaOH (50 mM, low in carbonate; Merck Ltd) in combination with 1mmol/L Zinc acetate was used as the mobile phase (1 ml/min, isocratic conditions). Column temp was maintained at 27°C. Sugars were detected using a Thermo Scientific UltiMate 3000 Series ECD (ECD-3000RS) with a gold working electrode and solid-state reference electrode.

[3] Playford RJ, MacDonald CE, Calnan DP, Floyd DN, Podas T, Johnson W, Wicks AC, Bashir O, Marchbank T. Co-administration of the health food supplement, bovine colostrum, reduces the acute non-steroidal anti-inflammatory drug-induced increase in intestinal permeability. Clin Sci (Lond). 2001 Jun;100(6):627-33. PMID: 11352778.

c. The potentials were set as follows:

	Potential (mV)	Duration (mS)
E1	150	500
E2	700	100
E3	-900	100
E4	150	10

d. Data analyses were performed using the ThermoFisher Scientific Dionex Chromeleon 7 chromatography data system. Typically, the mannitol peak eluted from the column at approx. 3 min, rhamnose at 4 min and lactulose at 16 min. Internal Standard used was melibiose, which eluted at 10 mins.

e. Data was presented as lactulose/rhamnose ratios to maintain consistency with previous studies[4] and the fact that, using the protocol described, the mannitol peak has been reported to be occasionally obscured by overlap from other urinary constituents. In addition, assessment of gut permeability using lactulose:rhamnose ratio as our index of intestinal injury, is a commonly used combination for assessing enteropathy.

Samples gifted for future research will be kept stored frozen indefinitely.

---

[4] Playford RJ, MacDonald CE, Calnan DP, Floyd DN, Podas T, Johnson W, Wicks AC, Bashir O, Marchbank T. Co-administration of the health food supplement, bovine colostrum, reduces the acute non-steroidal anti-inflammatory drug-induced increase in intestinal permeability. Clin Sci (Lond). 2001 Jun;100(6):627-33. PMID: 11352778.

e) Strategies adopted to increase recruitment during the study

During the study, we instigated several strategies to increase recruitment. These included:

- Promoting the study through the CICRA website (https://www.cicra.org/)
- Securing ethical approval for remote informed consent to minimise hospital visits during the COVID-19 pandemic
- Plan to enlist Manchester Children's Hospital, UK as a PIC but this was not progressed due to the COVID-19 restrictions
- Repeat screening of CYP who were initially ineligible due to unstable disease, a recent change in treatment or similar factors. As a result, 95 children were screened twice, 31 three times, 11 four times and 2 five times
- Emphasise to potential participants that they can exit the study at any time – e.g. after completing the 6 week double blind phase

f) Findings from qualitative research

Reasons for and decisions about taking part in the study

Nine young people talked about altruistic reasons for wanting to take part in the study. Typically, they talked of wanting to help other people, for example *"help [other kids]"*, although others talked of wanting to *"help with Crohn's really"* (FM007) *or "give something back"* (FM023). Three young people provided context for wanting to help in relation to their own experiences of milk treatments, one explained *"help other people 'cause it helped me so much when I was on the Modulen" (FM018*), another said *"I just wanted to change from the Modulen"* (FM023) and the other said:

> *"I wanted to be involved in it [research] just to help out other people and make it easier for them. When I had the milkshakes a few years ago I didn't find it that easy and had to have a tube and stuff like that so. I just wanted to make it easier for other people"* (FM008).

The young people reported that the study had been explained *"very well"* (FM009) and they understood the purpose of the study and what was involved with a typical response being *"I understood what I was doing"* (FM003). Their initial impression was that the study *"sounded fine"* (FM007), *"sounded okay"* (FM011), and thought *"it was quite interesting"* (FM008). Overall, the explanation of the study was reassuring and *"worth a try out"* (FM016), with one young person explaining:

> *"I didn't think I was doing something, like, extreme, I just thought I was just drinking something to see if it improves my health"* (FM003).

One young person said they were *"surprised because I never thought I'd do a medical trial, but I think I changed"* (FM015); this suggests that the study was described well and was not off-putting.
Some of the young people provided various levels of detail about the purpose of the study in their own words, for example, *"to help people like feel better" (FM016) "to find out if the special milk is a good option"* (FM0018) and:

> *"The study is trying to figure out if there's, not sort of medicine but along them lines, and to see if anything can ease the pain in any way. One hasn't got anything in, it's just normal milk isn't it? And then the other one is the one that they're going to use if it works"* (FM017).

Some also explained the crossover design with various degrees of success: one young person seemed to misunderstood that they would get first milk in the second six weeks as they said *"but then the second six weeks it was definitely the cow's milk"* (FM022) whereas another young person was much clearer:

> *"In clinic they explained in the first half we don't know what it is, what the milk is and then like in the second half, we know that it's definitely the like first milk"* (FM020).

One young person provided an explanation of the study based on their experience of drinking the milkshake for a week:

> *"If I was talking to someone thinking about taking part, I would just say to them that the milkshake is nice and there is no need to be afraid of drinking it, it's easy and only one glass a day so, it's not bad"* (FM008).

Some of the parents explained their reasons for wanting to take part in the study. Typically, this was because they were interested to find out if it would help their child:

> *I was really intrigued to find out if it would help him, so I was interested and you know, I wanted to just get on and do it really* (FM007).

The decision to participate was talked about as a joint decision, often underpinned by a sense of altruism, for example, *"I spoke to him about it showed him the letter and he was quite keen to take part, purely to help other people really"* (FM008). Other parents were more overtly informed by a desire to improve their child's health as *"[IBD is] such a horrible thing for them to have to have, anything that would help is important"* (FM022). One parent could see benefits to be part of a research study as her son would be monitored:

> *"I'm glad that he's been having his bloods and his weight. It was just nice to keep a check on him as well. So, we benefit as well as the research"* (FM007).

However, the decision to participate was not always straightforward with parents expressing some reservations about any potential negative impacts the trial might have on their child's health such as worries that *"it might cause another flare up…. we weren't sure if it would mess with her tummy"* (FM009). Another parent's explanation reflects how they weighed up the pros and cons balancing these with an altruistic impulse:

> *"Our first thoughts about the trial were a bit mixed – we didn't want to upset anything as he has just got well really. But on the flip side we thought there may be something good from this milk which would help him. Pros and cons really – and we have been so grateful to the hospital it enabled us to give something back"* (FM018).

It was clear that parents valued their children's opinions:

> *"He wasn't too keen initially but because he's been on liquid diets in the past and they've initially worked and then they haven't. Once I explained to him that this was something different and it may be a better substitute for what's out there at the moment, to give your bowel a rest, he agreed that it was worth taking part"* (FM016).

One parent noted that they were *"very proud that she [daughter] just took part in"* the study (FM009).

Acceptability of study design and processes

Overall, the processes associated with the study were acceptable to the young people with two talking about how they sustained their motivation to stay engaged in the study by thinking that *"if I stop then it'll just be pointless"* (FM016) and that sticking with the study would *"help people"* (FM015); the latter young person went on to say that:

> *"only once or twice across the whole time I thought I don't really want to do this anymore, but I think I was just having a bad day and it just felt a bit too much on top of everything else"* (FM015).

Four young people talked about study duration; three thought the length of time was OK, stating that it is *"all right, to be honest"* (FM023), *"I don't think it was like to long of a time period or anything like that"* (FM009) and that the *"six weeks to take the milk is about right and not too long"* (FM018). Only one young person felt that the duration was *"a little bit too long, if I was designing it I would make it probably three to four weeks"* (FM017).
Part of the willingness for parents to take part was that the design of the study was seen to be *"was pretty straight forward"* (FM015) and that taking part was generally seen as being *"easy to do, it hasn't took up much time to do"* (FM008) and *"fine"* (FM003, FM018, FM020). The trial team was described as *"fabulous…[as] they had was explained [study] really well… provided all the information we needed"* (FM018). One parent reported a sense of reassurance that the study was fair as:

> *"everyone gets a go of the special milk, the first part was randomised but everyone got a go so I thought it was interesting to know that he'll definitely get it as part of the trial"* (FM015).

The clarity about being able to opt out was reassuring for parents. One child became unwell and his milkshakes were paused for a few days before restarting and his parent explained:

> *"we did feel very able to say we didn't want to continue and the professor called us and was really nice and said that he wanted us to feel comfortable and no-one would mind if we [had] wanted to drop out"* (FM018).

The opportunity to be *"flexible"* (FM011) and to fit *"the hospital [study] visits round normal visits"* (FM009) and was seen as beneficial, not least because it avoided their child *"missing too much school"* (FM018). One parent noted that if this had not happened *"they probably wouldn't have taken part, because…we live nearly two hours away from the hospital"* (FM023). The benefits of the study being aligned to routine appointments for infusions or other reasons was that the research team:

> *"just used to take extra blood and things......for the research, so again it was pretty much the same as what we were doing anyway"* (FM011).

One parent explained that even though they *"had to do a few extra visits to the hospital to drop off samples"* that this was not a *"[not a] hassle [or] a big disruption into your routine or anything"* (FM015).

For parents of children who were used to taking milkshakes, the fact that the study was milkshake-based was seen as only a slight shift in routine, for example, "*he was on the Modulen anyway, so it's been - yeah, hasn't caused any inconvenience at all"* (FM023) *and "he's used to having [Modulen] every night"* (FM016). However, it was acknowledged that this might not be the case for all parents or children:

> *"so I'm just treating this new milk as a replacement for that [Fortini]. Maybe people who've never had milkshakes before, maybe they might feel differently about it"* (FM003).

Another parent noted:

> *"I suppose it's difficult if a child doesn't like that type of milk and things, but I wouldn't hesitate in recommending this type of trial to any parent or children"* (FM011).

The need to make up the milkshakes was not seen as off-putting, as one parent explained:

> *"I think you'd do anything for your child won't you so that didn't bother us. We just sailed through that and so yeah, I wasn't bothered about making it"* (FM007).

Acceptability of completing diaries, questionnaires, and interviews

Overall, the seven young people who commented on completing the diary thought it was acceptable, noting that it was *"okay"* (FM018, FM022), *"easy to do"* (FM007, FM023), and *"no hassle"* (FM008). Although the diary did not take long to complete *"like 2 minutes maybe, tops"* (FM015) it did need *"a bit of effort"* (FM018). One young person said they filled it in every night but also noted that *"my mum has to remind me a few times"* (FM008). A couple of young people said they sometimes forgot to complete it; this meant they *"did a couple of days at a time, 'cos the answers were quite similar, really"* (FM023) or *"filled in a few days at a time"* (FM017).

The questionnaires were mentioned by a three young people; one young person thought the questionnaires were *"alright…straightforward"* (FM007), another noted that the questionnaires were "*okay, easy, just ticking boxes – they were not difficult questions"* (FM018), whereas the other young person noted that the:

> *"questions on the questionnaire were a bit weird though. It was stuff like….'Do you think your Crohn's disease is going to affect you getting a boyfriend or girlfriend?' I just thought like, it's never crossed me mind! Why would anyone think that?"* (FM017).

Only one your person mentioned the interviews and they said that the *"interviews have been fine"* (FM008).
Some parents talked about the acceptability of the other aspects of data collection with a parent noting that the diaries were not a problem as they were *"really easy to fill in … takes two minutes of your time"* (FM016). Whilst one parent reported feeling reassured by the weekly check-in calls as they *"helped us feel less anxious about whether the milk was making him poorly at all"* (FM018) another parent said that although the calls were *"fine on a day off…. whether something like a quick online email, questionnaire or something"* (FM022) would be more convenient.

Acceptability of samples taken during the study

Eight young people talked about the acceptability of the samples that were taken as part of the study; of these, one explained they were *"not a fan of the samples"* (FM022). Three reported that they were used to having samples taken so these *"didn't really bother me"* (FM009, FM008, FM007). However, some young people reported that even though they were used to samples being taken, they *"do not like blood tests"* (FM023) and they still got *"stressed about [blood test but] manage to get on with [them]"* (FM018). Two young people warned that blood tests *"might put people off"* (FM023, FM018) from participating in the study. Having the samples taken alongside a routine hospital visit was perceived as being *"just easier…. than having to go some way to it"* (FM0123). Only one young person talked about the urine test being *"really annoying"* (FM017) and they said:

> *"I didn't like doing the urine sample as you have to mix things up and then you need to fast and I think that might put people off ….. I had to starve myself for four hours, I'm not used to that" (FM017).*

One young person struggled with the instructions because they could not *"read instructions really… the way it was saying you've got a mix it altogether"* (FM017). Another young person said that the *"samples were a bit confusing sometimes"* (FM020).
Overall, parents did not see the need for their children to provide stool and blood samples as particularly problematic as their children were used to providing samples; one parent explained that her son was no longer *"bothered [by stool samples], he just does it"* (FM007). However, although the children and parents accepted the need for the samples, for some children it did require them to be resilient or brave. One parent explained that her daughter *"wasn't very happy about the stool samples, it's just not the nicest thing for her to have to do but it wasn't that off-putting"* (FM022). Another parent noted that her:

> *"[son] was fine with the samples, but he does get really stressed with blood tests - but they were really helpful in making sure he had the tests at the same time so he didn't need to have extra blood tests" (FM018).*

Interest in the outcomes of this and future studies

Six young people provided comments about what they perceived as important outcomes; of these, three thought we had asked about *"all the important things"* (FM020) or could not *"think of anything else to be measured"* (FM022). One young person suggested we should ensure that we encouraged documentation of even *"small amounts of pain, probably like small niggles in my stomach".* One young person talked of linked outcomes "*the right nutrition and...not making me ill or anything"* (FM023). In terms of a future study, one young person questioned whether a study that focused solely on a once-a-day milkshake rather than a milk-only regime would be a *"true representation of real life"* or *"really show how milk is tolerated by young people"* (FM023).
Several parents expressed an interest in the overall study results, variously noting that they *"would really want to know the outcome of the study – overall – what has the trial found out what happens next. It is important for families to know"* (FM018) and they were *"interested to find out what's happening with it [especially] if it was rolled out nationally"* (FM016). One parent reported they were excited about her son's results and noted that:

> *"I think [son] has enjoyed it as well because he was excited to get on the scales and see how much he'd gone up in weight because he was only six and a half stone"* (FM007).

Acceptability of the milk

Many of the young people were used to a regime of taking different milks and so taking the milkshake daily was part of their normal routine. One parent noted that "s*he [daughter] used to take Modulen anyway, so it's pretty much the same sort of thing really*" (FM011).

> *"She used to have a Modulen milkshake every day anyway…so for her the only difference was that she wasn't having that, she was having a different milkshake"* (FM022, Mother).

In some cases, it was the dislike of these other dietary milk drinks and wondering if there was a better alternative which had led the young people to join the First Milk study, for example "*He just hates the Modulen and wants to find another milk*" (FM018) and "*I just wanted to change from the Modulen*" (FM023).

Flavour

Many of the young people and parents compared the flavour of the First Milk drink to other dietary milks they had taken previously.

> *"He's drunk these milkshakes that are First Milk ones, better than he drunk, you know, the Nutricia ones that he had previously. He likes this type of drink more than he liked that kind of drink"* (FM003, Mother).

One young person preferred the taste of the First Milk shake to Modulen, noting "*It was easier to drink than the Modulen. I liked the flavour better*" (FM023).
In contrast, two of the young people preferred the taste of Modulen to the First Milk shake "*I think I prefer the Modulen that I had before*" (FM020) and one mother described how this meant that her daughter was pleased to come to the end of the study:

> *"She didn't like them as much as the Modulen...so I think she was quite happy she's gone back onto the Modulen now"* (FM022, Mother).

Some of the young people described the differences in tastes between the placebo and the First Milk powder and which one they preferred. One young person described the First Milk powder as "*tasting worse than the other one [placebo*]" and "*tasting stronger, more of off milk*" (FM008). One young person particularly disliked the taste of the First Milk drink:

> *"I didn't like the taste or texture of this milkshake [First Milk] at all – it was difficult to get it all down. It tasted horrible as I was drinking it"* (FM018).

In contrast some young people preferred the taste of the First Milk shake to the placebo.

> *"This milkshake [First Milk} is definitely nicer than the other one [placebo]. It just tastes more like a milkshake"* (FM016).

Even the young people who did not like the taste of the First Milk shake, still preferred it to the placebo "*I didn't like it [the First Milk], but I didn't really mind it, it was better than the first one [placebo]*" (FM020). One young person described how the taste of First Milk just mixed up with milk was fine

> *"It [First Milk] doesn't taste that bad, actually. I thought it would taste pretty bad but…it tastes of milk"* (FM015).

Some young people described an aftertaste from the First Milk drink "*there is a bit of an after taste that I don't like*" (FM008) and this taste could persist for some time after drinking the shake, "*I get an aftertaste at the back of my throat for about 20 mins after I have drunk it. I have a glass of water*" (FM018).
Young people were asked that if they were to design their own milkshakes, how they would improve it. One young person stated that they would alter the First Milk shake by "*making it less vanillery, less of an aftertaste of vanilla*" (FM007)

Disguising the flavour

Many of the young people added flavours to the First Milk drink to improve the taste as "*when you just have it with water and nothing else, that tastes horrible*" (FM003). Another young person who was having First Milk for both six-week periods, described how they:

> *"Put it in ice cream and milk, strawberry ice cream. A few scoops to get the taste away of the powder"* (FM007).

Many of the young people felt that with something added *"it just tastes like I'm drinking, like, a normal milkshake"* (FM003) and that by *"putting vanilla essence in it or chocolate Nesquik then it just tastes like a milkshake that you get in the shop"* (FM017). The mother of one young person discussed how the taste of First Milk meant they had to use different flavours to disguise it:

> *"The second milk [First Milk] wasn't kind of nice, so we had to kind of be a bit more inventive about disguising it really compared to the first 6 weeks [placebo]"* (FM008).

Many of the young people used similar techniques and flavour combinations with the placebo or drew on experiences of disguising the taste of previous dietary milkshakes.

Texture

There was a wide variability in how the young people and their parents described the texture of the First Milk shake. The texture of the First Milk drink was described by some young people as "*grainy or gritty at the bottom*" (FM003) which could be unpleasant. Sometimes the whole of the milkshake was described as gritty, for example, "*no matter how much I mix it, on the top it's still got a bit of bits in it*" (FM017). However, young people who were taking the placebo also described the same "*powdery, gritty*" (FM008) powder at the bottom on the shake.
The powdery texture was often discussed alongside the First Milk shake being *"a lot thicker and quite lumpy and with a sediment at the bottom"* (FM018 mother). The sediment at the bottom was disliked by quite a few of the young people:

> *"This milkshake [First Milk] is a bit thicker and I do not like it as much as the last one [placebo], there is a bit at the bottom which is a bit sludgy and yucky"* (FM018).

The *"grittiness"*, *"lumpiness*" and *"globbiness*" of the shake could be reduced by blending the shake:

> *"We used a fork to mix the first milkshake at first, but it was very lumpy, it was disgusting really. We couldn't get the lumps out so we borrowed a blender off somebody and we're blending it and it's a lot smoother and it's a lot easier…"* (FM009).

Using a blender was described as making the First Milk shake much *"better and easier to drink"* (FM016) and *"much better than stirring it [First Milk] with a spoon"* (FM020) and *"blending it made it less gritty at the bottom"* (FM023).
Whilst using a blender improved the palatability of the First Milk shake, one mother said having to use and wash out a blender daily was a disadvantage of this dietary shake compared to alternatives:

> *"Modulen, we just mix with a whisk and it goes really smooth, really quickly. So this stuff [First Milk] was really bitty and I had an old blender and I used that but it didn't really make any difference. So, he did complain initially, 'Oh it's got bits in, I don't like it'. So, I went out and bought another blender and that seemed to do the trick. But to be honest, if you've got to use your blender every time, I know it sounds trivial but just getting the blender out, and then having to blend it and then wash the blender… I don't have to do that with the Modulen you see"* (FM016).

Some young people described the First Milk shake as "*frothy*" (FM007), "*foamier*" (FM008) and "*thinner and frothier*" (FM022) than the placebo. This resulted in some of the young people preferring the texture of the placebo as the First Milk was "*a bit too frothy on the top*" (FM011).

As described above, many of the families learnt that blending the First Milk powder made it more palatable; however, this could also make it frothier:

> *"if it were to be given as a milk drink it didn't mix that great and we had to blend it and blending it made it really frothy"* (FM008).

The texture of the milkshake was a really important factor in how easy young people found it to follow the regimen and participate in the trial, as this young person noted:

> *"It's kind of like frothy like a milkshake. It's not like, too watery. It's not like water, it's like a milkshake like texture. the thickness of it was maybe quite clumpy, It was like, it didn't really mix very well. Towards the end I was managing to drink it but like it was getting a bit like oh, it's this drink again. I managed it alright, it was just kind of getting a bit annoying taking it at the end"* (FM016).

This was echoed by another young person who was struggling to continue to take First Milk during the six-week period:

> *"I didn't drink it right down to the bottom as there was a bit of like froth left. Six weeks felt a bit longer on this shake [First milk], I was feeling I have had enough a little bit near the end of this one, but not really the first one [placebo]..."* (FM022).

Two of the young people noted that if they were to *"design my own milkshake, I would make it thinner"* (FM011) and "I'd prefer it to be a little bit thinner" (FM015).
Some young people noted the unpleasant texture of the placebo milkshake and described some "*grittiness*" and "*sediment at the bottom*". However, the texture of the First Milk shake seemed pronounced.

Smell

Some young people and parents detected a noticeable difference in the smell associated with the First Milk powder and milkshake. The following quote is a graphic description:

> *"When you open the bag, to be honest, it had a horrible smell. The smell actually made me feel a bit sick and I did think to myself when it's mixed, we mixed it with water, if it's mixed up and it smells like this, he's not going to drink it"* (FM016, Mother).

The smell was noted by both parents and young people:

> *"This one smells like a strange milk – so I have to use a straw as it is a bit off putting"* (FM018).

Detecting differences between the different milks

Many of the young people who had been taking First Milk for both six-week periods recognised that the milk they had been taking "*was the same, I think I had the same both times*" (FM009). However, some who had taken First Milk for both periods described how they thought their milk had changed "*This one tastes a bit more milky but better than the*

last one, Like milkier, like a creamier kind of taste" (FM011), and other young people describing that even though the "*texture was the same, the taste was oh so slightly different, I preferred the taste of the second one" (*FM015). The following young person who had taken First Milk throughout discussed how they had also detected a difference in the milks.

> *"I could definitely taste a difference in the milks, like the first one wasn't the actual milk 'cos the taste was different, the first one just tasted like normal milk but the second one tasted like me other one, you know the Modulen I used to drink, it just tasted like that really. The second one tasted like, thicker, it was a bit frothy, but like it wasn't bad"* (FM017).

Some young people detected a difference in the texture between the two milkshakes they had, even when they had taken First Milk for both time periods, such as "*it was a bit more gritty than the first one*" (FM023).

The young people who had swopped over between the placebo and the First Milk shake had detected the difference. The following young person had noticed a difference in the colour of the powder.

> *"I knew which one was the real one because you get the real one on the second six weeks and it looks different, the powder looked different to the first one. The powder, it's more yellowy. and the first six one, the pretend one, was white"* (FM016).

Perceptions of impact on symptom management

The sample of young people interviewed was small and so caution needs to be applied in over interpreting the qualitative interview data linked to any reported changes in symptoms. Some young people and their parents noted no differences in any symptoms over the period of the study and when trying the First Milk "*It didn't make any difference to how I felt*" (FM011). Many of the young people were clinically 'well' and symptom free when they joined the study. The following mother described how they had not noticed any difference in her daughter's condition over the course of the study.

> *"We didn't really notice a difference in how she is I don't think so, I mean she's very stable, touch wood. At the moment, so, she is in remission with her Crohn's. I wouldn't say that has been any different with her on this milk than it was you know, when she was on the Modulen really* (FM022, Mother).

There were several of the young people who reported positive effects of the First Milk shake on any symptoms they had or their general well-being. One young person who received First Milk for the whole of the study period stated that *"I think it's working yeah. I think it's working"* (FM007). In some young people improvements were described as small such as "*not feeling as bloated after I ate food or anything like that"* (FM009) or a boost of energy:

> *"Normally I'm always drained because I suffer with iron deficiency. But it's given me a little bit of a boost, not a lot but I can notice it. There was a few symptoms just going the toilet and that. Yeah, the milkshake's made it a little bit better - going less often"* (FM017).

In other young people improvements were described as more profound:

"He has put seven kilo on so we know now that he has more energy and he can put weight on with [milkshake] so I've got the address now to get more. I'm going to give him it." (FM007)

Some of the improvements were subjective, whilst others were described as noticeable on clinical tests.

"His stool sample, his calprotectin levels had gone down to twenty which is the lowest they've been for a long time. The time before I think they were fifty or sixty so I don't know whether that's anything to do with the drink or it's his infusion but it seems coincidental that they'd gone down" (FM016).

In one instance the First Milk shake was reported as linked to increasing clinical symptoms

"It [First Milk] made me feel a bit stomach achey afterwards a few times" (FM018).

In some cases young people described a change in symptoms when they had received First Milk for the whole period of the study "*I think I felt better on the first one I think* but t*he second one I feel like it gave me a bit more energy"* (FM007).

Similarly, some young people described how they felt '*full*' after drinking the milkshakes whether this was the placebo or the First Milk milkshake:

"I started to feel fed up with it about fourth week-ish.in. Since it filled me up, I couldn't really have anything else to eat for a while after" (FM020).

Figure S1: Trial profile

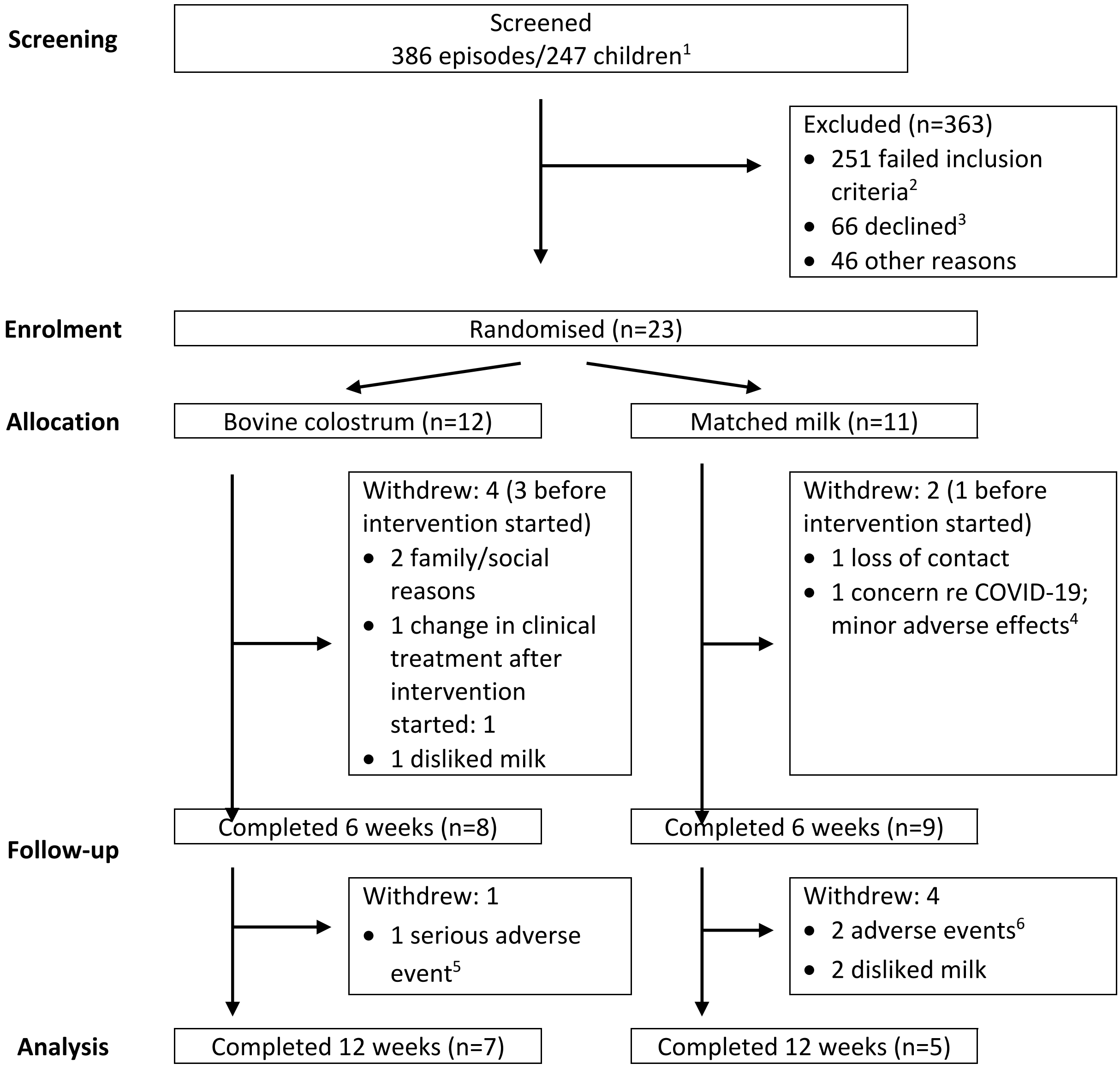

Notes

1. 95 children were screened twice, 31 three times, 11 four times and 2 five times
2. Main reasons for failing inclusion criteria were other diagnosis or Crohn's disease not confirmed (78), already receiving nutritional supplement (64), clinically unstable or recent change in treatment (61), outside of age range or transitioned/being transitioned to adult care (27), newly diagnosed (8), allergic/intolerant to cows' milk (7)
3. Main reasons for declines were no reason or felt unable to comply with the study requirements (24), dislikes milk/nutritional supplements (24), currently stable and unwilling to change management (18)
4. Child experienced catarrh, sore throat and wind
5. *Campylobacter* enteritis requiring hospital admission
6. One child had worsening of eczema; one child had a relapse of oro-facial granulomatosis

Table S1: Diaries completed during the blinded phase (weeks 1-6)

Variable	Bovine colostrum	Placebo milk	P value
**Week 2**			
• No. diaries completed	7	7	-
• No. days information reported median (range)	7 (6-7)	7 (7-7)	-
• 100% adherence; No. (%)	5 (71.4)	6 (85.7)	1.0*
**Week 6**			
• No. diaries completed	8	8	
• No. days information reported (median; range)	7 (7-7)	7 (6-7)	
• 100% adherence; No. (%)	5 (62.5)	5 (62.5)	1.0
Feeling after taking the milk No. CYP	7	4	-
No. days information reported (median; range)	7 (5-7)	6.5 (5-7)	-
Feeling score (median; IQR, range)	0.58 (0.50-0.79) 0.50-1.0	0.7 (0.50-0.98) 0.50-1.0	1.0*
Did the milk make you feel better? (no. %)			
• definitely felt better	2 (33.3)	1 (14.3)	0.56*
• no difference	4 (66.7)	6 (85.7)	

*Fisher's Exact Test

Table S2: Routine haematology and biochemistry according to intervention group

Variable	Baseline No. Median (IQR) First milk	Baseline Placebo	Change in variable Week 0-6 No. Median (IQR) First milk	Change in variable Week 0-6 Placebo	P value	Change weeks 1-12; FM group No. Median (IQR)	Change in all children after 6 weeks FM No. Median (IQR)
Haematology							
Hb (g/L)	11<br>129.0<br>(125.0 to 146.0)	10<br>134.0<br>(125.5 to 137.8)	8<br>-2.5<br>(-6.0 to 8.5)	9<br>4.0<br>(0.0 to 6.5)	0.64	8<br>-3.0<br>(-9.75 to 6.75)	13<br>-1.0<br>(-6.5 to 14.2)
MCV (fl)	11<br>84.0<br>(83.0 to 85.0)	10<br>324.5<br>(81.8 to 91.0)	8<br>0.5<br>(-1.0 to 1.75)	9<br>0.0<br>(-3.0 to 1.0)	0.34	8<br>1.0<br>(-1.75 to 2.75)	13<br>-1.0<br>(-3.0 to 1.0)
Platelet count ($x 10^9$/l)	11<br>274.0<br>(219 .0 to 344.0)	10<br>86.5<br>(251.5 to 360.5)	7<br>-20.0<br>(-43.0 to -9.0)	9<br>-16.0<br>(-40.5 to 1.0)	1.0	8<br>-28.5<br>(-50.5 to 14.5)	12<br>-34.0<br>(-52.75 to 23.4)
WCC ($X10^9$/L)	11<br>6.51<br>(5.02 to 8.00)	10<br>5.79<br>(4.46 to 7.74)	8<br>0.53<br>(-0.40 to 1.17)	9<br>0.09<br>(-0.33 to 0.19)	0.35	8<br>0.20<br>(-0.12 to 0.85)	13<br>0.45<br>(-0.05 to 1.15)
Neutrophils ($x10^9$/L)	11<br>3.10<br>(2.52 to 5.16)	10<br>3.73<br>(1.88 to 4.69)	8<br>0.94<br>(0.08 to 1.6)	9<br>-0.02<br>(-4.10 to 0.23)	0.057	8<br>0.34<br>(-0.73 to 0.82)	13<br>0.23<br>(-0.34 to 0.94)
Lymphocytes ($x10^9$/L)	11<br>2.31<br>(1.38 to 2.86)	10<br>1.56<br>(0.99 to 1.80)	8<br>-0.08<br>(-0.29 to 0.15)	9<br>0.14<br>(-0.17 to 0.63)	0.64	8<br>0.03<br>(-0.07 to 0.13)	13<br>0.07<br>(-0.19 to 0.38)
Eosinophils ($x10^9$/L)	11<br>0.17<br>(0.08 to 0.25)	10<br>0.14<br>(0.11 to 0.26)	8<br>-0.02<br>(-0.07 to 0.01)	9<br>-0.02<br>(-0.06 to 0.05)	1.0	8<br>-0.01<br>(-0.08 to 0.13)	13<br>-0.01<br>(-0.06 to 0.03)

Basophils (x10^9/L)	11<br>0.04<br>(0.03 to 0.05)	10<br>0.045<br>(0.03 to 0.053)	8<br>0.0<br>(-0.02 to 0.01)	9<br>0.0<br>(-0.02 to 0.01)	1.0	8<br>0.01<br>(-0.15 to 0.02)	13<br>0.00<br>(-0.02 to 0.01)
Erythrocyte sedimentation rate (mm/hour)	11<br>8.0<br>(6.0 to 10.0)	10<br>7.0<br>(4.8 to 9.3)	8<br>0.0<br>(-2.5 to 0.75)	9<br>0.0<br>(-1.0 to 2.0)	1.0	7<br>1.0<br>(-1.0 to 6.0)	13<br>0.00<br>(-4.0 to 1.0)
Biochemistry							
Plasma urea (mmol/L)	11<br>3.50<br>(3.20 to 4.20)	10<br>3.25<br>(1.98 to 3.98)	8<br>-0.05<br>(-0.53 to 0.68)	9<br>0.1<br>(-0.6 to 0.7)	0.64	8<br>0.35<br>(-0.275 to 0.8)	13<br>-0.10<br>(-0.65to 0.90)
Plasma creatinine (µmol/L)	11<br>48.0<br>(40.0 to 62.0)	10<br>55<br>(46.8 to 65.5)	8<br>0.0<br>(-5.0 to 2.0)	9<br>-1.0<br>(-4.5 to 0.5)	1.0	8<br>-3.0<br>(-5.75 to 6.5)	13<br>0.0<br>(-3.5 to 2.0)
Plasma calcium (mmol/L)	11<br>2.35<br>(2.32 to 2.41)	9<br>2.34<br>(2.24 to 2.39)	8<br>0.0<br>(-0.45 to 0.02)	8<br>0.0<br>(-0.04 to 0.09)	1.0	8<br>-0.05<br>(-0.14 to 0.07)	12<br>0.0<br>(-0.02 to 0.05)
Plasma phosphate (mmol/L)	11<br>1.27<br>(1.22 to 1.37)	9<br>1.33<br>(1.16 to 1.60)	8<br>0.0<br>(-0.13 to 0.19)	8<br>-0.55<br>(-.15 to 0.03)	0.62	8<br>-0.12<br>(-0.21 to 0.20)	12<br>0.05<br>(-0.07 to 0.19)
Plasma ALP (IU/L)	11<br>143.0<br>(85.0 to 194.0)	10<br>249.0<br>(108.5 to 319.5)	8<br>-2.5<br>(-13.3 to 6.5)	9<br>-1.0<br>(-14.0 to 13.5)	1.0	8<br>-1.0<br>(-21.5 to 16.5)	13<br>0.00<br>(-17.0 to 12.5)
Plasma AST (IU/L)	11<br>19.0<br>(17.0 to 22.0)	10<br>21.0<br>(15.5 to 25.8)	8<br>0.5<br>(-0.33 to 7.5)	9<br>0.0<br>(-2.0 to 1.0)	1.0	8<br>-3.0<br>(-8.75 to 0.00)	13<br>-1.0<br>(-4.0 to 2.0)
Plasma ALT (IU/L)	11<br>10.0<br>(9.0 to 13.0)	10<br>13.0<br>(10.5 to 19.5)	8<br>2.0<br>(-2.25 to 5.0)	9<br>0.0<br>(-1.5 to 0.5)	0.15	8<br>2.5<br>(-0.25 to 6.0)	13<br>0.0<br>(-3.0 to 4.0)
Plasma total bilirubin (g/L)	11<br>11.0<br>(5.0 to 15.0)	10<br>14.5<br>(8.0 to 19.0)	8<br>0.0<br>(-3.8 to 1.5)	9<br>-1.0<br>(-5.0 to 3.0)	0.35	8<br>-0.5<br>(-0.8 to 1.8)	13<br>0.0<br>(-4.0 to 1.5)

Plasma total protein (g/L)	11<br>76.0<br>(75.0 to 80.0)	10<br>75.5<br>(70.3 to 76.8)		8<br>1.0<br>(-2.3 to 3.8)	9<br>-1.0<br>(-3.0 to 1.5)	0.35		8<br>-0.5<br>(-2.8 to 2.8)	13<br>1.0<br>(-2.5 to 3.5)
Plasma Albumin (g/L)	11<br>44.0<br>(40.4 to 45.0)	10<br>44.0<br>(42.0 to 45.0)		8<br>0.5<br>(-1.0 to 2.8)	9<br>0.0<br>(-1.0 to 1.0)	0.64		8<br>0.5<br>(-1.0 to 2.8)	13<br>1.0<br>(-1.0 to 2.5)

Table S3: Multiplex immunoassay results according to intervention and follow-up*

	MIP1-alpha (CCL3)	IL-1 beta	IL-4	IP-10 (CXCL10)	IL-6	IL-8 (CXCL8)	IL-10	IL-12 P70	IL-13	IL-17A (CTLA-8)	IFN-gamma	GM-CSF	TNF-alpha	MIP-1 beta (CCL4)	IFN-alpha	MCP-1 (CCL2)	CD62P	**IL-1 alpha**	**ICAM-1**	**CD62E**
Baseline																				
Bovine colostrum n=4																				
Mean	33.67	4.67	15.36	6.32	54.50	2.01	9.21	7.7	10.11	5.70	9.40	33.18	38.77	25.13	3.43	78.82	12853.7	1.48	95625.8	16978.0
SD	17.70	4.27	12.34	2.68	60.43	1.67	12.92	12.0	10.38	5.18	8.59	29.01	62.54	10.68	3.91	14.36	10995.6	1.04	60963.8	8524.2
Placebo n=4																				
Mean	32.77	29.69	32.44	6.33	118.79	6.26	17.28	86.2	28.78	32.08	69.36	130.39	64.95	20.11	8.49	69.36	44796.8	0.59	110930.7	17804.1
SD	26.97	58.46	62.31	2.26	220.16	10.51	31.83	144.8	46.32	56.65	125.15	223.42	119.75	6.97	14.82	39.29	37951.9	0.25	59235.7	6538.4
Total n=8																				
Mean	33.16	17.18	23.90	6.33	97.36	4.13	13.25	46.96	20.78	18.89	43.66	81.78	51.86	22.62	5.96	74.09	32019.6	1.04	103278.3	17391.0
SD	21.64	40.64	42.57	2.29	175.83	7.33	22.90	101.5	34.76	39.82	94.25	156.37	89.54	8.77	10.39	27.85	32504.0	0.85	56245.4	7046.8
Week 6																				
Bovine colostrum n=2																				
Mean	38.82	2.36	6.57	5.84	23.51	1.88	20.69	7.97	14.52	3.66	8.67	23.21	3.54	26.41	3.09	37.57	24404.4	1.03	95393.2	10120.7
SD		0.65	0.91	4.81	16.60	2.27	24.88	0.77		1.81	1.08	6.41		15.94	1.20	19.89		0.63	67767.0	1215.3
Placebo n=4																				
Mean	31.88	19.14	24.12	6.94	110.63	5.69	12.09	39.65	18.84	18.18	49.29	87.41	42.70	22.76	6.69	58.07	37655.7	0.49	105308.6	17583.7
SD	28.63	36.64	45.11	2.24	187.78	8.55	21.36	68.20	23.79	28.67	83.44	145.04	76.53	4.75	11.17	37.78	36816.0	0.37	60769.8	6519.5
Week 12																				
Bovine colostrum n=2																				
Mean	31.81	1.57	6.46	4.39	29.73	1.54	15.30	6.54	10.16	3.53	7.18	12.10	60.29	25.07	2.62	101.82	20970.6	0.87	108176.8	11160.3
SD	10.61	1.55	6.72	1.03		0.62	20.63	7.27	1.49	0.85	2.45	9.30	80.25	8.76	1.34	75.48	1023.8	0.15	85991.8	3061.4

*All analyte concentrations are reported in pg/m
